# Remote sensing and spectroscopy of lichens

**DOI:** 10.1002/ece3.11110

**Published:** 2024-03-03

**Authors:** Miina Rautiainen, Nea Kuusinen, Titta Majasalmi

**Affiliations:** ^1^ Department of Built Environment Aalto University School of Engineering Espoo Finland; ^2^ Department of Forest Sciences University of Helsinki Helsinki Finland

**Keywords:** airborne, biocrust, cryptogam, lichen, reflectance, satellite image, spectra, UAV

## Abstract

Lichens are combinations of two symbiotic organisms, a green alga or cyanobacterium and a fungus. They grow in nearly all terrestrial ecosystems and survive in habitats, which are very dry or cold, or too poor in nutrients to maintain vegetation growth. Because lichens grow on visible surfaces and exhibit spectral properties, which are clearly different from, for example, vegetation, it is possible to distinguish them in remote sensing data. In this first systematic review article on remote sensing of lichens, we analyze and summarize which lichen species or genera, and in which habitats and geographical regions, have been remotely sensed, and which remote sensing or spectroscopic technologies have been used. We found that laboratory or in situ measured spectra of over 70 lichen species have been reported to date. We show that studies on remote sensing of lichens fall under seven broad themes: (1) collection of lichen spectra for quantification of lichen species or characteristics, (2) pollution monitoring with lichens as ecological indicators, (3) geological and lithological mapping, (4) desert and dryland monitoring, (5) animal habitat monitoring, (6) land cover or vegetation mapping, and (7) surface energy budget modeling.

## Graphical abstract

We analyzed and summarized which lichen species or genera, and in which habitats and geographical regions, have been remotely sensed, and which remote sensing or spectroscopic technologies have been used. We found that laboratory or in situ measured spectra of over 70 lichen species have been reported to date. We showed that studies on remote sensing of lichens fall under seven broad ecological themes.

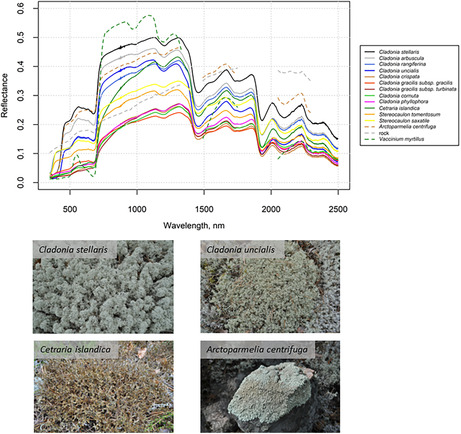

## 1 INTRODUCTION

**FIGURE 1:**
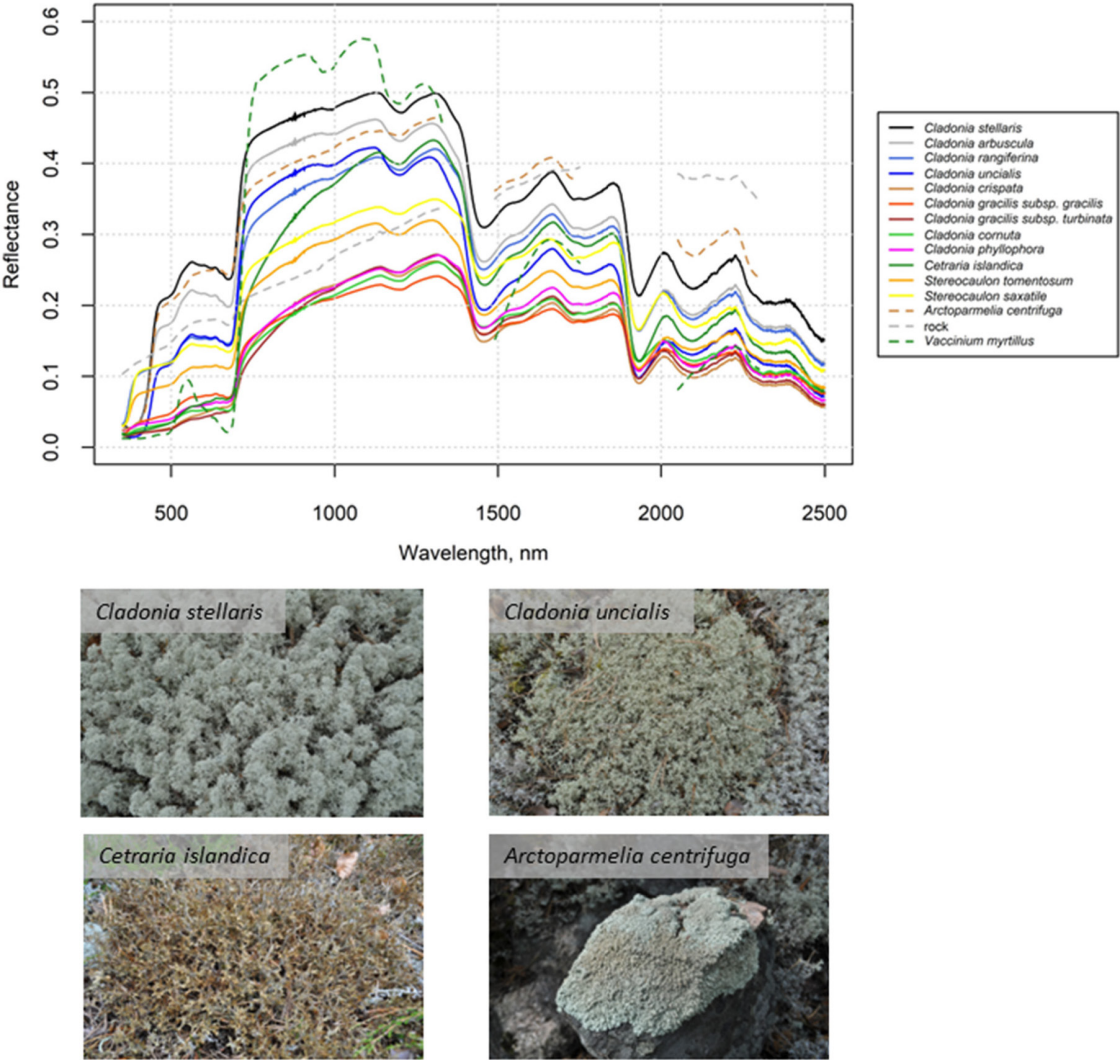
Examples of lichens and their reflectance spectra (mean spectrum for each species), contrasted with reflectance spectra of typical surfaces that lichens are mixed with in remote sensing applications (green vegetation: *Vaccinium myrtillus*, rock: weathered granite and granodiorite). The study sites and measurement protocols used for collecting lichen spectra have been reported in Kuusinen et al. (2020) (for solid lines, in laboratory conditions) and Kuusinen et al. (2023) (for dashed lines, in field conditions).

Lichens are combinations of two symbiotic organisms, a green alga or cyanobacterium and a fungus (Figure 1). They grow in nearly all terrestrial ecosystems and survive in habitats which are very dry or cold, or too poor in nutrients to maintain vegetation growth. This also means that lichens can form biocrusts on versatile surfaces such as rocks and soil, or grow as epiphytes on tree bark or even on industrial structures. The number of lichen species has been estimated to be ca. 18,000 (Sipman & Aptroot, 2001), yet there are numerous unresolved taxonomic problems related to lichens because the concept of species is not always clearly defined, and tropical areas have been underexplored in terms of lichen diversity (Feuerer & Hawksworth, 2007). Also, the global coverage and biomass of lichens is unknown, lichens grow in nearly all of the Earth's terrestrial areas, and especially in high latitude areas, lichen species richness is higher than that of vascular plant species (Nash, 2008).

Lichens have multiple roles in our environment. For example, they provide food for reindeer and caribou (e.g., Bernes et al., 2015), have a major role on the energy budget and albedo of high latitude areas (e.g., Bernier et al., 2011), form extensive biocrusts in arid areas (e.g., Ferrenberg et al., 2015), and serve as ecological indicators of air pollution (e.g., Conti & Cecchetti, 2001). All these applications have led to the development methods for measuring and monitoring lichen community structures using remote sensing and spectroscopic data. Because lichens exhibit spectral properties which are clearly different from, for example, vegetation and rock surfaces (Figure 1), it is possible to distinguish them in remote sensing data.

In this paper, we review the currently existing scientific literature related to remote sensing of lichens. More specifically, we report which lichen species, and in which habitats and geographical regions, have been remotely sensed, and which remote sensing or spectroscopic technologies have been used. Based on the literature review, we characterize trends and recurring research themes, and point out future perspectives for remote sensing of lichens.

## 2 LITERATURE SEARCH

We conducted a literature search in Web of Science using the search words “lichen” AND “remote sensing.” The search was extended to the entire content of the articles, that is, it was not limited to the title, abstract and keywords of the articles. We included only papers published in English in peer‐reviewed journals until March 2023 (cut‐off‐date for article search: 31 March 2023). This search resulted in 278 articles. We prereviewed the articles to identify which of them focused on remote sensing of lichens. Our criteria for including an article were that (1) the paper contained explicit results based on spectral in situ or laboratory data or remote sensing data of lichens or lichen‐covered areas, and (2) results contained and discussed information directly related to lichens. After the prereview phase, 148 articles were left for the review. In addition to these articles, we included 5 other articles or reports which were relevant to the topic but did not show up in the literature search. These were original findings or pilot studies of lichen spectroscopy that had been published as conference proceedings or technical reports several decades ago. A complete list of the reviewed 153 articles (which form the basis for the figures and tables in this article) is provided in the Appendix S1 even though all of the articles are not cited directly in this article's text.

For the articles that were included in the review, we recorded and analyzed the following information: geographical location of the study site and extent of sampling scheme, names of studied lichen species (according to their taxonomic status when the original study was published) and their growing habitats, lichen properties that had been measured, name of remote sensing (or handheld/laboratory) sensor, and motivation, goals and main conclusions of the study. During the analysis of the articles, it became evident that it was not possible to record all this information for all studies. For example, many articles lacked information on the names of lichen species because the goal of the study had been, for example, to map lichen cover and not species. Surprisingly, some articles also lacked detailed information on the location of study site (coordinates), description of the growing environment (land cover) of the lichens, or the exact name of the remote sensing sensor that had been used. Most articles also did not contain data on lichen properties (e.g., cover or biomass) but focused merely on the presence or absence of lichens in the study area.

## 3 LICHEN SPECIES, CHARACTERISTICS, AND HABITATS IN REMOTE SENSING STUDIES

Globally, species belonging to 52 different lichen genera were mentioned in the articles (Tables 1 and 2). However, it should be noted that only ~35% of the articles included names of lichen species or genera, and often the information on the species or genera was not explicitly used to analyze or interpret any results. The reasons for not including species information are likely related to the difficulties in identifying lichen species without expert help and, in some applications, the fact that species‐specific information was not relevant. Based on the list of lichen species that have been mentioned in the reviewed articles (Table 1), there is a bias toward species that form large growths (mats), occur commonly, or which are relatively easily identifiable.

**TABLE 1:** Lichen species and/or genera in the reviewed articles (Appendix S1) in different continents. Note that (i) only ~35% of the reviewed articles mentioned explicit information on lichen species/genus, and (ii) there were no articles reporting lichen species from the Asian part of Russia, and thus Russia is included in full as part of Europe in this table.

Africa (2 genera)
*Psora* sp., *Diploschistes* sp.
Antarctica (13–14 species/12 genera)
*Buellia* sp., *Caloplaca* sp., *Caloplaca regalis*, *Haematomma erythroma*, *Himantormia lugubris*, *Lecanora physciella*, *Leptogium puberulum*, *Leptogium puberulum*, *Mastodia tessellata*, *Nostoc commune*, *Physconia muscigena*, *Rhizoplaca melanophthalma*, *Usnea* sp., *Xanthoria elegans*
Asia (4 species/4 genera)
*Catapyrenium crustosum*, *Psora decipiens*, *Ramalina lacera*, *Xanthoparmelia desertorum*
Europe (62–66 species^a^/34 genera)
*Acarospora nodulosa*, *Acarospora* sp., *Alectoria ochroleuca*, *Buellia epigea*, *Buellia zoharyi*, *Cetraria hepatizon*, *Cetraria islandica* subsp. *islandica*, *Cetraria islandica*, *Cetraria juniperina*, *Cetraria nivalis*, *Cladina/Cladonia arbuscula*, *Cladina/Cladonia rangiferina*, *Cladina/Cladonia stellaris*, *Cladina/Cladonia uncialis*, *Cladonia arbuscula* subsp. *squarrosa*, *Cladonia cornuta* subsp. *cornuta*, *Cladonia crispata var. crispata*, *Cladonia crispata*, *Cladonia furcata*, *Cladonia gracilis* subsp. *gracilis*, *Cladonia gracilis* subsp. *turbinata*, *Cladonia mitis*, *Cladonia phyllophora*, *Cladonia uncialis* subsp. *uncialis*, *Collema cristatum*, *Cornicularia divergens*, *Diploschistes diacapsis*, *Diploschistes diacapsis*, *Diploschistes* spp., *Endocarpon pusillum*, *Flavocetraria nivalis*, *Fulgensia desertorum*, *Fulgensia fulgens*, *Haematomma ventosum*, *Hypogymnia physodes*, *Lecidea lithophila*, *Lepraria crassissima*, *Lepraria sp*., *Lobaria amplissima*, *Melanelia hepatizon*, *Nephroma arctica*, *Ophioparma ventosa*, *Parmelia caperata*, *Parmelia olivacea*, *Parmelia perlata*, *Peltigera horizontalis*, *Peltigera leucophelbia*, *Placidium* sp., *Placynthium nigrum*, *Pseudephebe pubescens*, *Psora decipiens*, *Rhizocarpon* sp., *Squamarina lentigera*, *Stereocaulon* sp., *Stereocaulon saxatile*, *Stereocaulon tomentosum*, *Sticta sylvatica*, *Teloschistes lacunosus*, *Teloschistes* sp., *Thamnolia vermicularis*, *Toninia sedifolia*, *Umbilicaria arctica*, *Umbilicaria hirsuta*, *Umbilicaria pustulata*, *Umbilicaria rigida*, *Umbilicaria spodochroa*, *Umbilicaria vellea*, *Xanthoria parmetina*
North America (72 species/30 genera)
*Alectoria nigricans*, *Alectoria ochroleuca*, *Arctoparmelia centrifuga*, *Arctoparmelia incurve*, *Arctoparmelia separata*, *Asahinea chrysantha*, *Aspicilia cinerea*, *Caloplaca cernia*, *Candelariella aurella*, *Candelaria concolor*, *Catapyrenium squamulosum*, *Cetraria cucullata*, *Cetraria cucullate*, *Cetraria nivalis*, *Dimelaena oriena*, *Cladina/Cladonia mitis*, *Cladina/Cladonia rangiferina*, *Cladina/Cladonia stellaris*, *Cladonia amaurocraea*, *Cladonia arbuscula*, *Cladonia bacilliformis*, *Cladonia bellidiflora*, *Cladonia borealis*, *Cladonia botrytis*, *Cladonia carneola*, *Cladonia chlorophaea*, *Cladonia coccifera*, *Cladonia cyanipes*, *Cladonia deformis*, *Cladonia metacorallifera*, *Cladonia pleurota*, *Cladonia scabriuscula*, *Cladonia squamosa*, *Cladonia ulphurina*, *Cladonia transcendens*, *Cladonia uncialis*, *Collema coccophorum*, *Collema tenax*, *Dactylina arctica*, *Flavocetraria cucullata*, *Flavocetraria miniscula*, *Flavocetraria nivalis*, *Hypogymnia physodes*, *Lecanora dispersa*, *Lecanora muralis*, *Melanelia disjuncta*, *Melanelia septentrionalis*, *Melanelia sorediata*, *Melanalia stygia*, *Nephroma arcticum*, *Ochrolechia frigida*, *Parmeliopsis ambigua*, *Peltula patellata*, *Pertusaria* sp., *Phaeophyscia orbicularis*, *Physcia aipolia*, *Physcia dubia*, *Physcia phaea*, *Physcia caesia*, *Placynthium asperllum*, *Placynthium nigrum*, *Rhizocarpon bolanderi*, *Rhizocarpon geminatum*, *Rhizocarpon geographicum*, *Rhizocarpon geographicum*, *Rinodina turfacea*, *Scoliciosporum chloroccum*, *Sphaerophorus globosus*, *Thamnolia subuliformis*, *Umbilicaria deusta*, *Umbilicaria muehlenbergii*, *Umbilicaria torrefacta*
South America (7 species/7 genera)
*Acarospora* cf. *gypsi‐deserti*, *Buellia* sp., *Caloplaca santessoniana*, *Placidium* cf. *velebiticum*, *Pleopsidium chlorophanum*, *Rinodina* sp., *Stereocaulon* sp.

^a^ The number varies because species classification has changed after the studies were conducted.

**TABLE 2:** Summary of lichen species which have had their spectral properties measured.

Species	Reference
*Acarospora* cf. *gypsi‐deserti*	Lehnert et al. (2018)
*Alectoria ochroleuca*	Gauslaa (1984), Petzold and Goward (1988)
*Arctoparmelia centrifuga*	Morison et al. (2014)
*Aspicilia cinerea*	Bechtel et al. (2002)
*Buellia* sp.	Casanovas et al. (2015)
*Caloplaca* sp.	Casanovas et al. (2015)
*Caloplaca santessoniana*	Lehnert et al. (2018)
*Candelariella aurella*	Morison et al. (2014)
*Candelaria concolor*	Morison et al. (2014)
*Cetraria ericetorum*	Petzold and Goward (1988)
*Cetraria hepatizon*	Gauslaa (1984)
*Cetraria islandica*	Granlund et al. (2018), Kaasalainen and Rautiainen (2005), Kuusinen et al. (2020, 2023), Nordberg and Allard (2014)
*Cetraria juniperina*	Gauslaa (1984)
*Cetraria nivalis*	Gauslaa (1984), Petzold and Goward (1988), Rees et al. (2004)
*Cladonia arbuscula*	Granlund et al. (2018), Kaasalainen and Rautiainen (2005), Kuusinen et al. (2020, 2023), Nordberg and Allard (2014), Rees et al. (2004)
*Cladonia crispata*	Kuusinen et al. (2020, 2023), Rees et al. (2004)
*Cladonia cornuta* subsp. *cornuta*	Kuusinen et al. (2020)
*Cladonia gracilis* subsp. *gracilis*	Kuusinen et al. (2020)
*Cladonia gracilis* subsp. *turbinata*	Kuusinen et al. (2020)
*Cladonia furcata*	Kaasalainen and Rautiainen (2005)
*Cladonia phyllophora*	Kuusinen et al. (2020)
*Cladonia rangiferina*	Granlund et al. (2018), Kaasalainen and Rautiainen (2005), Kuusinen et al. (2020, 2023), Neta et al. (2010), Neta et al. (2011), Nordberg and Allard (2014), Peltoniemi et al. (2005)
*Cladonia stellaris*	Bubier et al. (1997), Gauslaa (1984), Granlund et al. (2018), Kaasalainen and Rautiainen (2005), Kushida et al. (2004), Kuusinen et al. (2020, 2023), Neta et al. (2010, 2011), Nordberg and Allard (2014), Peltoniemi et al. (2005), Petzold and Goward (1988), Solheim et al. (2000)
*Cladonia uncialis*	Granlund et al. (2018), Kuusinen et al. (2020, 2023), Rees et al. (2004)
*Cornicularia divergens*	Gauslaa (1984)
*Dimelaena oriena*	Morison et al. (2014)
*Flavocetraria nivalis*	Granlund et al. (2018), Solheim et al. (2000)
*Haematomma ventosum*	Gauslaa (1984)
*Hypogymnia physodes*	Kaasalainen and Rautiainen (2005)
*Lecanora dispersa*	Morison et al. (2014)
*Lecanora muralis*	Morison et al. (2014)
*Lecidea lithophila*	Rees et al. (2004)
*Leptogium puberulum*	Milos et al. (2018)
*Lobaria amplissima*	Gauslaa (1984)
*Melanelia disjuncta*	Morison et al. (2014)
*Melanelia hepatizon*	Rees et al. (2004)
*Melania stygia*	Morison et al. (2014)
*Nephroma arctica*	Gauslaa (1984), Rees et al. (2004)
*Nostoc commune*	Milos et al. (2018)
*Ophioparma ventosa*	Rees et al. (2004)
*Parmelia caperata*	Gauslaa (1984)
*Parmelia olivacea*	Gauslaa (1984)
*Parmelia perlata*	Gauslaa (1984)
*Peltigera horizontalis*	Gauslaa (1984)
*Peltigera leucophelbia*	Gauslaa (1984)
*Physcia caesia*	Morison et al. (2014)
*Physconia muscigena*	Milos et al. (2018)
*Placidium* cf. *velebiticum*	Lehnert et al. (2018)
*Placynthium asperllum*	Morison et al. (2014)
*Placynthium nigrum*	Morison et al. (2014)
*Pseudephebe pubescens*	Gauslaa (1984)
*Rhizocarpon* sp.	Rees et al. (2004)
*Rhizocarpon bolanderi*	Bechtel et al. (2002)
*Rhizocarpon geminatum*	Bechtel et al. (2002)
*Rhizocarpon geographicum*	Bechtel et al. (2002), Morison et al. (2014)
*Rhizoplaca melanophthalma*	Milos et al. (2018)
*Rinodina* sp.	Lehnert et al. (2018)
*Stereocaulon* sp.	Granlund et al. (2018), Rees et al. (2004)
*Stereocaulon paschale*	Petzold and Goward (1988)
*Stereocaulon saxatile*	Kuusinen et al. (2020), Nordberg and Allard (2014)
*Stereocaulon tomentosum*	Kuusinen et al. (2020)
*Sticta sylvatica*	Gauslaa (1984)
*Thamnolia vermicularis*	Gauslaa (1984)
*Umbilicaria arctica*	Gauslaa (1984)
*Umbilicaria deusta*	Morison et al. (2014)
*Umbilicaria hirsuta*	Gauslaa (1984)
*Umbilicaria pustulata*	Gauslaa (1984)
*Umbilicaria rigida*	Gauslaa (1984)
*Umbilicaria spodochroa*	Gauslaa (1984)
*Umbilicaria torrefacta*	Bechtel et al. (2002)
*Umbilicaria vellea*	Gauslaa (1984)
*Usnea* sp.	Casanovas et al. (2015)
*Xanthoria elegans*	Milos et al. (2018)
*Xanthoria parmetina*	Gauslaa (1984)

**FIGURE 2:**
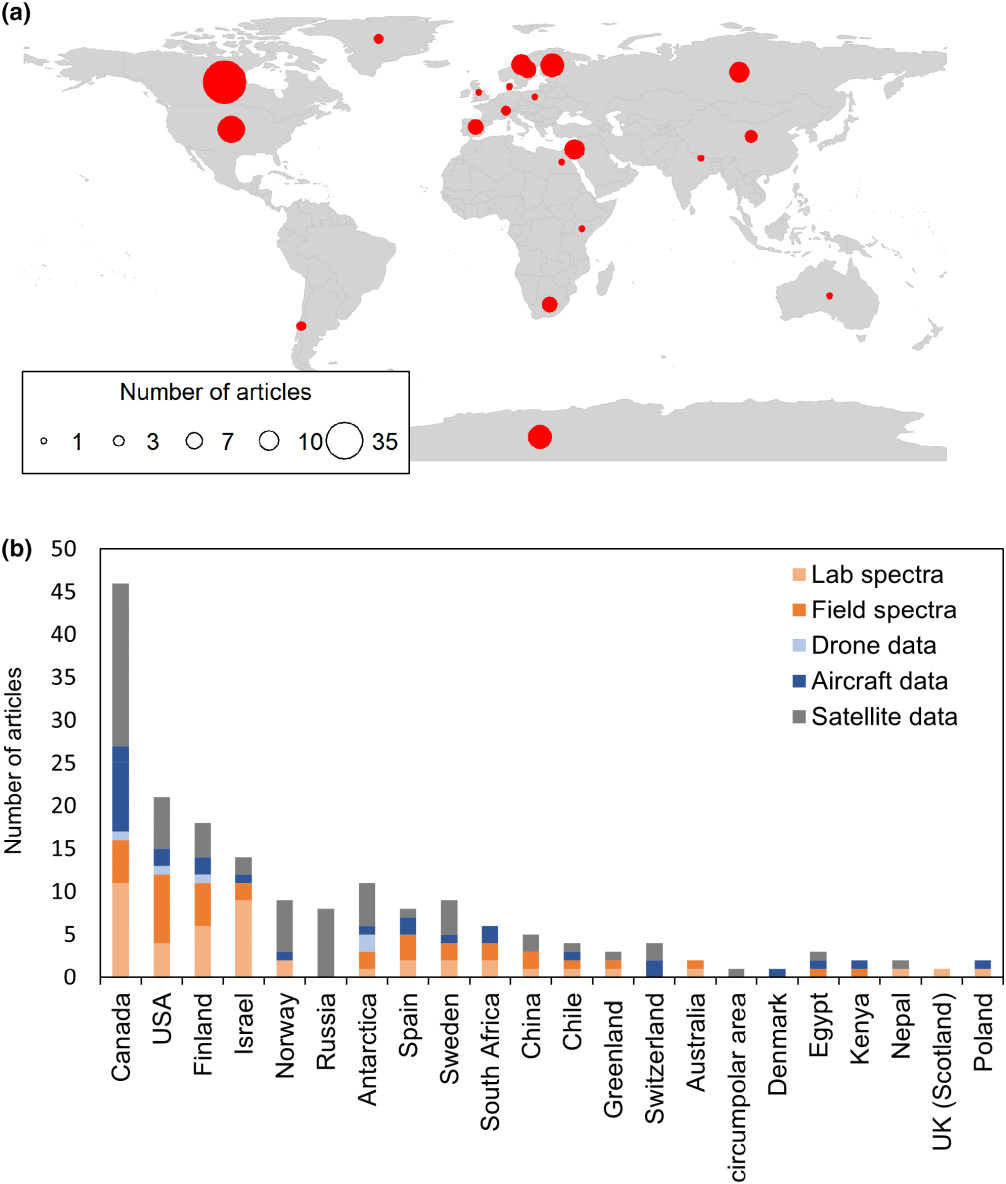
Geographical distribution of studies related to remote sensing of lichens. (a) Approximate locations of study areas in articles related to remote sensing of lichens. (b) Numbers of articles reporting remote sensing of lichens using different remote sensing platforms.

High latitude regions (comprising the boreal, tundra and Arctic/Antarctic zones) represented 75% of the published articles related to remote sensing of lichens. Approximately 40% of all studies on remote sensing of lichens have been conducted in North American areas (Figure 2). In addition, there have been three other active regions for lichen studies: Fennoscandia (i.e., Finland, Norway, Sweden), Antarctica and Israel (Figure 2). Studies based on lichen data from Africa, Asia, Australia, and South America are currently scarce.

**FIGURE 3:**
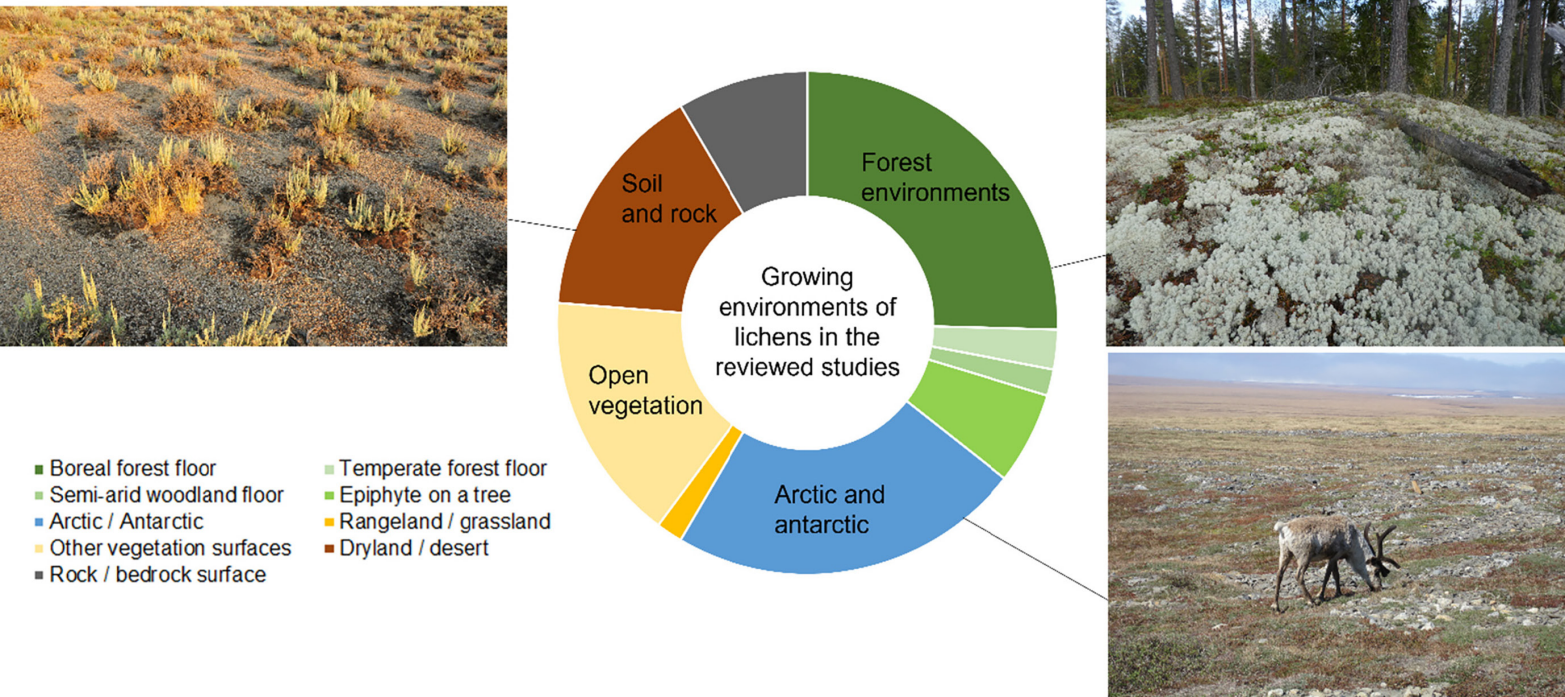
The proportions of growing habitats of lichens present in the reviewed studies. The photographs show examples of lichens in different environments. Upper left corner: Biological soil crust (Photo credit: United States Fish and Wildlife Service (Tom Koerner), licensed under the Creative Commons Attribution 2.0 Generic license). Upper right corner: Lichens forming large mats on boreal forest floor. Lower right corner: A caribou grazing on lichens (Photo credit: Bering Land Bridge National Preserve, licensed under the Creative Commons Attribution 2.0 Generic license).

Overall, approximately one‐third of the growing habitats for the studied lichens were in forest ecosystems, one‐third in other types of vegetation ecosystems (e.g., open tundra or rangelands), and one‐third on bare soil, sand, or rock surfaces (Figure 3). Approximately 50% of the studies had recorded some in situ information on lichen properties instead of merely noting the presence or absence of lichens in an area or on a surface. The most common lichen property that had been measured or visually estimated was lichen cover (in ~25% of the reviewed studies, also called e.g., cover percent, fractional cover, projection cover, coverage or lichen ground cover in the reviewed articles). In addition, lichen biomass, height, moisture content, chemical properties, or other ecophysiological indicators had each been measured in ~5% of all studies.

## 4 TYPES OF REMOTE SENSING DATA IN LICHEN‐RELATED STUDIES

**FIGURE 4:**
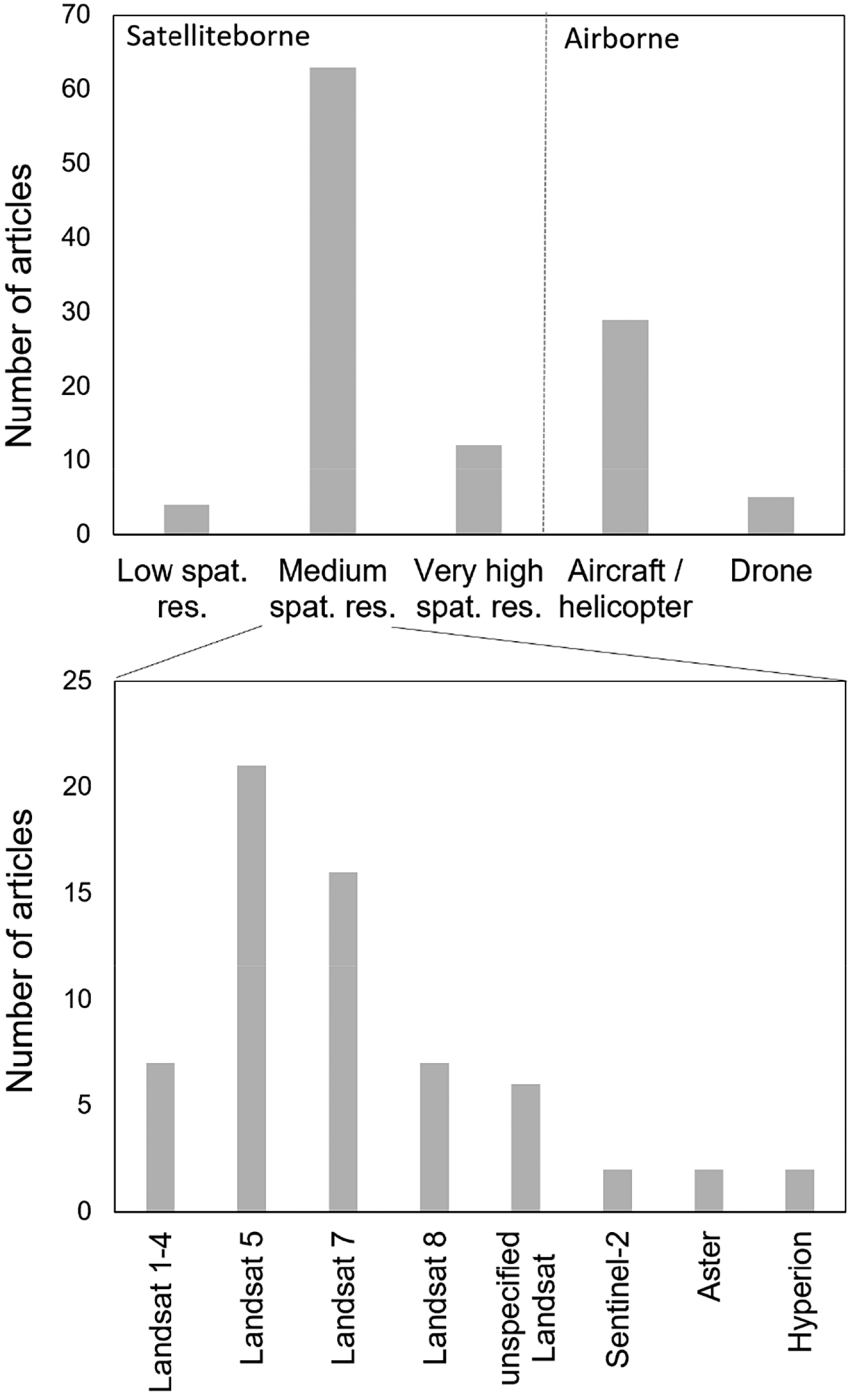
Platforms and sensors (applying data from visible, near‐infrared and shortwave infrared spectral regions) used in peer‐reviewed articles reporting studies on remote sensing of lichens before April 2023. The upper figure shows numbers of articles using different satellite and airborne platforms, and the lower figure shows in detail the numbers of articles utilizing different medium spatial satellite sensors (spatial resolution 10–100 m) to sense lichens. Other sensor types are not shown as a separate figure because the number of studies per sensor was often only one or two. We report them in the following. Coarse spatial resolution satellite sensors included AVHRR and MODIS (spatial resolution > 100 m). Very‐high spatial resolution satellite sensors were World‐view‐2, Quickbird, KOMPSAT‐3 and SPOT‐5 (spatial resolution < 10 m). Airborne sensors included single studies utilizing traditional aerial photos, CASI and/or SASI, SEBASS, AISA Eagle/Hawk/Dual, APEX, AVIRIS, Probe‐1, Riegl LMS‐680i, Optech ALTM3100, or Leica ALS50‐II. Drone sensors included unnamed RGB cameras, Ocean Optics FLAME, Canon EOS M6 and S110 NIR adapted, Parrot Sequoia, and Senop Rikola Hyperspectral Imager.

Remote sensing of lichens has been conducted at various spatial scales using different types of sensors (Figure 4). Approximately half of the published studies have used optical satellite data, one‐fourth of the studies have used airborne (aircraft or helicopter based) data and under 5% of studies have utilized data acquired by sensors on unoccupied aerial vehicles (UAV). Nearly all studies have applied passive optical remote sensing techniques, but there are a couple of exceptions of using active remote sensing technologies where LiDAR (Kaasalainen & Rautiainen, 2005; Korpela, 2008; Moeslund et al., 2019) or an active normalized difference vegetation index (NDVI) sensor (Erlandsson et al., 2023) have been employed. Spectral reference data (or close‐range remote sensing data) of lichen samples or entire communities have been collected in laboratory or field conditions in about 30% and 40% of the articles, respectively.

In satellite remote sensing, medium spatial resolution sensors have been commonly used, with multispectral data from Landsat mission sensors being the most frequently applied (Figure 4). For example, in applications related to monitoring lichens in reindeer or caribou habitats or lichens as bioindicators of pollution, mainly Landsat data have been used. Multispectral satellite data from Sentinel‐2 MSI and ASTER, and hyperspectral satellite data from Hyperion have been used in a much lesser extent in all applications. Very high spatial resolution satellite data (from KOMPSAT‐3, SPOT‐5, Worldview‐2, Quickbird) have been applied in under 10% of the studies, and mainly in the highest latitudes. In airborne studies, conventional aerial images have been the most common data source but also hyperspectral sensors (e.g., CASI, SASI, APEX, AVIRIS, and AISA family sensors) have been used in about 10% of the studies. Most studies applying airborne hyperspectral data have been related to geological or lithological mapping or monitoring of dry areas. In laboratory and field measurements, ASD FieldSpec series spectrometers (~30 studies), GER series spectrometers (11 studies), Li‐COR Li‐1800 (~10 studies) and Ocean Optics S2000 (~5 studies) have been the most commonly used instruments for collecting spectral reference data of lichens. In addition, 22 other spectral measurement devices have been applied, typically in single studies.

There are also some geographical trends in the use of different remote sensing techniques in lichen related studies (Figure 2b). Whereas satellite and airborne remote sensing data have been utilized in nearly all regions, the largest number of close‐range remote sensing studies where laboratory or field spectra of lichens have been measured have been conducted in northern areas (Canada, USA, and Finland), and UAV‐based studies have also been limited to high latitude areas (Canada, USA, Finland, and Antarctica).

## 5 RECURRING THEMES IN STUDIES ON REMOTE SENSING OF LICHENS

### 5.1 General trends in remote sensing of lichens

**FIGURE 5:**
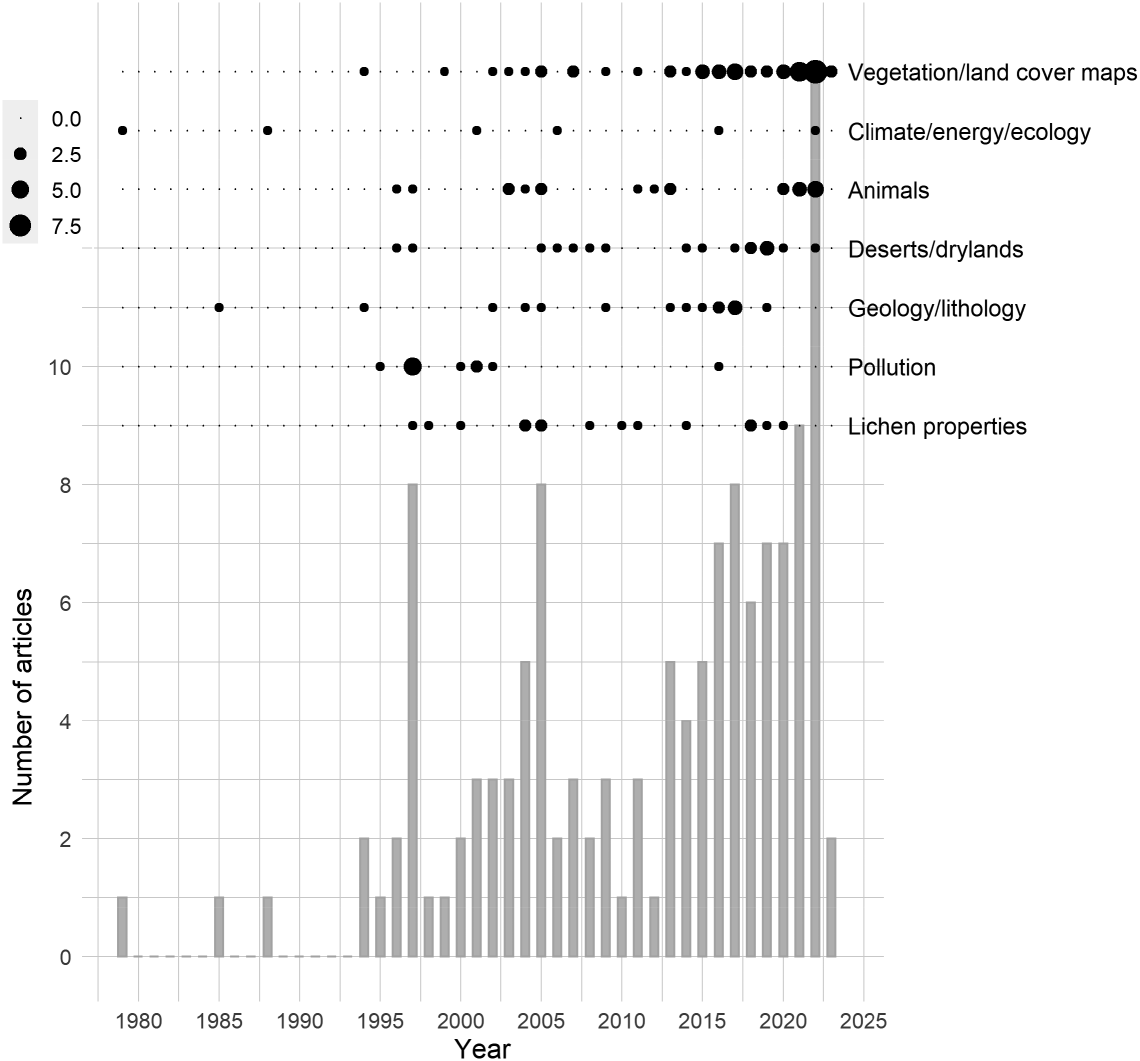
Trends in research related to remote sensing of lichens: how the number of published articles has developed (lower figure) and which themes the articles have focused on during each year (upper figure). The size of the circle (upper figure) refers to the number of published articles.

The first paper related to remote sensing (spectroscopy) of lichens to our knowledge was published in 1947 (Krinov, 1947) as a small part of a compilation work on the spectral properties of natural surfaces. However, before the mid‐1990s, there were only a few studies on remote sensing or spectral properties of lichens (Figure 5). Lichens have gradually grabbed the interest of the remote sensing community, and approximately 50% of the studies on lichens have been published within the past 10 years. The studies on remote sensing of lichens fall under seven broad themes: (1) spectral measurements for lichen identification and characterization, (2) pollution monitoring with lichens as ecological indicators, (3) geological and lithological mapping, (4) desert and dryland monitoring, (5) animal habitat monitoring, (6) extensive land cover or vegetation mapping, and (7) energy and carbon budget modeling.

Early studies, published in 1970s and 1980s, were related to lichen spectra, energy and carbon cycle modeling, and geological and lithological mapping (Figure 5). During the 1990s, the range of topics in remote of lichens widened considerably also to include quantification of lichen properties, and monitoring of animal habitats, drylands, and deserts. A popular theme in the late 1990s was related to the use of lichens in pollution monitoring, especially in Russia and Israel. In the beginning of the new millennium, the pollution monitoring theme continued still for a few more years alongside all the other themes. As global vegetation and land cover products became readily available, a clear increase in the number of studies reporting also results related to lichen covered areas was observed. In the early 2010s, a boom in the number of geological and lithological mapping papers occurred, and it was followed by an increase in the number of studies related to detailed quantification of lichen properties and applications in dryland and desert monitoring. In the next subsections, we will review in more detail studies related to these seven themes.

### 5.2 Spectral measurements for lichen identification and characterization

The earliest documentation on the general spectral properties of lichens was part of compilation reports on the reflectance properties of natural surfaces based on field spectroscopy (Krinov, 1947; Steiner & Gutermann, 1966) and did not include detailed descriptions of the measurement conditions or environments. Since 1984, laboratory or in situ measured spectra of over 70 lichen species have been published (Table 2), excluding pooled species spectra and spectra of unidentified lichens. Often, the motivation for measuring and publishing lichen spectra has been the need to investigate and understand the spectral properties of lichens so that the information could be further used to discriminate between lichen species or lichens from other substrates, or to estimate the response of lichens' spectral reflectance to environmental change. Limitations and uncertainties in the reported lichen spectra are related to measurement conditions: often information on lichen moisture content or a qualitative description of wetness/dryness of lichens and illumination conditions are missing.

Intra‐ and interspecific differences in lichen spectra have been studied by Gauslaa (1984), Ager and Milton (1987), Petzold and Goward (1988), Bechtel et al. (2002), Rees et al. (2004), Morison et al. (2014), and Kuusinen et al. (2020). Some of these studies also include in‐depth discussion about the possible reasons for variation in lichens' spectral characteristics, including the apparent color of lichens, lichen substances (secondary compounds produced by the mycobiont) composition, habitat (sunlit, shaded), moisture content, and growth form. A synthesis of these studies (see also Figure 1) shows that there are notable differences in the spectral properties of lichen species. For example, different *Cladonia* sp. lichens can have up to twofold differences in their reflectances in the near‐infrared region in similar measurement conditions (Kuusinen et al., 2020).

Gauslaa (1984) and Rees et al. (2004) suggested that different lichen species could be best separated using the near‐infrared region while Kuusinen et al. (2020) found the interspecific variation in lichens' spectra to be strongest in the ultraviolet and visible spectral regions. However, the spectral indices suggested for discrimination of some lichen species typically include spectral bands from the visible to shortwave infrared (Bechtel et al., 2002; Kuusinen et al., 2020). Additionally, Rees et al. (2004) observed the shortwave infrared region beyond water absorption at 1940 nm to be useful for discrimination between fruticose and crustose lichens.

The anisotropy of lichens' reflectance spectra has been studied by Solheim et al. (2000), Kaasalainen and Rautiainen (2005), Peltoniemi et al. (2005), and Kuusinen et al. (2020). All these studies observed that lichens scatter strongly in the illumination direction. Hence, this should be accounted for when interpreting remote sensing data (Kuusinen et al., 2023). This anisotropy information has been successfully exploited in a study that tested the use of intensity data from airborne discrete‐return LiDAR to map forest floor *Cladina* (*Cladonia*) cover (Korpela, 2008).

The possibility to separate lichens from vascular plants, mosses, or rocks has been discussed in a few studies that have collected lichen spectra. The spectra of lichens have been found to deviate most from those of vascular plants or mosses in the visible and near‐infrared spectral regions (Ager & Milton, 1987; Bubier et al., 1997; Petzold & Goward, 1988) (see also Figure 1). Bechtel et al. (2002) and Zhang et al. (2005) suggested to use a single (normalized) lichen endmember in the shortwave infrared region to separate different lichen species from rock. Ager and Milton (1987) also noticed absorption features occurring mainly in the shortwave infrared to be useful for recognizing lichen presence on rocks.

Lichens' water status is directly dependent on the environmental conditions (Nash, 2008), and the water content of lichens can change within minutes due to rain (Larson, 1987). Thus, relating lichens' reflectance spectra to their moisture content has been motivated by monitoring short‐term changes in local hydrologic conditions (Neta et al., 2010, 2011) or the photosynthetic status of lichens (Lehnert et al., 2018; Milos et al., 2018). For instance, Lehnert et al. (2018) noticed that the recovery in photosynthesis after watering two crustose chlorolichen species could be detected from the water absorption feature at 1420 nm. The response of lichen spectra to their changing moisture content has been observed to vary between species and even between studies. However, the most prominent effect of drying is generally to increase lichen reflectance in the shortwave infrared spectral range (Gauslaa, 1984; Granlund et al., 2018; Kuusinen et al., 2020; Lehnert et al., 2018; Neta et al., 2010, 2011; Nordberg & Allard, 2014; Rees et al., 2004). In the visible and near‐infrared regions, on the other hand, the differences between moist and dry lichens have been reported to vary between species (e.g., Kuusinen et al., 2020). Thus, understanding variation related to moisture content is critical in remote sensing and climate modeling applications of lichens. From a practical remote sensing perspective, spectral indices for estimating lichens' moisture content have been tested and developed by Neta et al. (2010, 2011), Granlund et al. (2018) and Milos et al. (2018).

### 5.3 Lichens as bioindicators in pollution monitoring

The idea of using lichens as bioindicators of air pollution was brought forward already in the 19th century by a leading lichenologist, William Nylander (Vitikainen, 2001), but using changes in the spectral properties of lichens to monitor pollution levels is a much more recent idea. In the late 1990s and early 2000s, a set of articles was published by Garty and his team on how air pollution influences, for example, the spectral properties of epiphyte lichens in trees in an industrial region and a national park forest area in Israel. Based on laboratory measurements, they analyzed how pollution in the lichens' habitat was related to the lichens' spectral reflectance, as well as photosynthesis and chlorophyll integrity (Garty, Tamir, et al., 2001), various physiological parameters (Garty, Cohen, Kloog, & Karnieli, 1997; Garty, Karnieli, Wolfson, et al., 1997; Garty, Kloog, Cohen, et al., 1997), and production of stress‐ethylene (Garty, Kloog, Wolfson, et al., 1997). The spectral response measurements were limited to examining the normalized difference vegetation index (NDVI). From a remote sensing perspective, the authors concluded that NDVI can detect early signs of pollutant‐induced stress for an epiphyte lichen species (*Ramalina lacera*, previously also known as *R. duriaei*).

A more detailed study on signatures of heavy metal complexes in lichen reflectance spectra was carried out in the proximity of a nickel smelter in Canada (Regan et al., 2016). Elemental concentrations of primary metals emitted by the smelter (Ni, Cu, Fe, Pb, Cd) and the reflectance spectra (350–2500 nm) of samples from 11 lichen species (located at different distances from the smelter) were measured in a laboratory. The study showed that, across lichen species, there were no spectral differences or absorption features attributable to specific metal complexes in lichens. The authors concluded that including multiple lichen species in such an analysis may have complicated interpreting the results because different lichen species may interact with airborne pollutants in different ways, and that undetermined microenvironmental effects may also have influenced the results. Overall, the results indicated that within‐species, within‐genus and spatial (within‐site) variation in lichen spectra can be so large that significantly more extensive sampling would need to be conducted to make more certain conclusions about the connections between heavy metal concentrations and spectral properties of lichens.

Geographically extensive, satellite‐based monitoring of the impact of pollution on lichens has mainly been carried out in northern Russia (e.g., Kola peninsula, Vorkuta) and areas in northern Norway (e.g., Varanger peninsula) using classification of Landsat MSS and TM data. These studies have been motivated by one of the largest sources of air pollutants in the Arctic area, the mining and metallurgical industry of the Kola Peninsula. In all the studies, field data on lichens (and vegetation) for large areas have been linked to satellite images to show that the damage of lichen/vegetation communities caused by atmospheric pollution can be assessed through satellite data (Rees & Williams, 1997; Tømmervik et al., 1995, 1998; Virtanen et al., 2002). The significant step forward shown by all these studies was that including Landsat data allowed a more spatially explicit analysis of the effects of pollution than the traditional in situ monitoring networks in the areas. Even though Tømmervik et al. (1998) suggested already 25 years ago that similar studies should be carried out using future remote sensing data sets which have higher spatial, spectral, and temporal resolutions, such studies have not been published to our knowledge in the context of lichen monitoring. This is perhaps because the topicality of the research theme in the area has gradually diminished due to the decrease in pollutants in the region after the 1990s (Paatero et al., 2008).

### 5.4 Role of lichens in geological and lithological mapping

Lichens growing on rock outcrops in nonrandom patterns, pose challenges for identifying minerals in geological mapping. This is especially a challenge in high latitude areas where lichens are abundant because they are well adapted to extreme environmental conditions. The mixing of spectral features of lichens and rocks can result in difficulties to identify spectral absorption features of minerals because lichens influence the spectral features of rocks notably. In the vast field of remote sensing applications in geology, the analysis of spectral properties of lichens forms a very small niche and covers fewer than 20 published articles. Most studies have been conducted in Canada but there are also a few from e.g., Greenland, Antarctica, Spain, Poland, Norway, and Scotland.

Laboratory spectroscopy studies have been conducted to understand how lichen cover influences the spectral properties of various minerals at wavelengths from approximately 400 to 2500 nm (e.g., Bechtel et al., 2002; Laakso et al., 2016; Morison et al., 2014; Rollin et al., 1994; Salehi et al., 2017; Zhang et al., 2005). The presence of lichens has been shown to influence specific absorption features of rocks as well as their spectral signatures' overall shape (Rollin et al., 1994). Already the earliest work demonstrated that lichen cover can either increase or decrease the spectral reflectance or even have no effect on the spectral properties of a rock in the wavelength region 400–1100 nm (Satterwhite et al., 1985). In a quantitative study covering a more extensive wavelength region (400–2500 nm), a 30% lichen cover on flat rocks (slate, hornfels) and a 60%–80% lichen cover on granite was shown to block the spectral signature of the rock (Ager & Milton, 1987). On the other hand, rock surfaces can be viewed to interfere with the discrimination or mapping of lichen species (Morison et al., 2014)—this highlights that the interference to the interpretation of the spectral signal can be viewed to be caused either by lichens or rocks, depending on the application. In the reviewed papers, lichen species had been identified in less than 50% of the articles related to geological applications, indicating that the presence or cover percentage of lichens on rocks was more important information than other qualities of the lichens.

The goal of the laboratory studies has often been to provide the scientific basis for future hyperspectral remote sensing applications, usually conducted at local or regional scale at either visible, near infrared and shortwave infrared (390–2400 nm) (e.g., Laakso et al., 2016) or longwave infrared (3–14 μm) (Feng et al., 2013) regions. Different forms of spectral unmixing have been the most common analysis method for separating components contributing to the spectral signal of a lichen‐substrate/rock mixture (e.g., Morison et al., 2014; Rivard et al., 2009; Zhang et al., 2004, 2005). The relatively good performance of linear mixture models in lichen/rock mixtures is based on the high optical thickness of the lichen layer (i.e., no photons pass through it), and thus, the spectral reflectance of lichen versus rock is linearly weighted with their respective cover (Bechtel et al., 2002). The basic understanding of spectral properties of lichens has been applied in, for example, the development of a spectral index that can been used to monitor the spatial–temporal development of lichen cover on lava surfaces (Li et al., 2015).

### 5.5 Monitoring lichens and biocrusts in deserts and drylands

Drylands around the world are often covered by biocrusts, and one component of biocrusts is lichens. The biocrusts have a critical role in for example, protecting arid surfaces from erosion and controlling their water regimes, and thus changes in biocrust cover and characteristics are linked to, for example, desertification processes. In this section, we use the term “biocrust” rather than lichen, because many of the reviewed studies reporting results from arid and semiarid regions do not make a clear distinction in reporting results for different types of biocrusts.

Basic reflectance characteristics of biocrusts were reported in the 1980s as part of geological applications (Ager & Milton, 1987; Jacobberger, 1989). Later, in situ or laboratory spectral data on biocrusts from arid and semiarid areas have been collected in several studies—either to support the interpretation of remote sensing data or to comprehend the spectral characteristics of biocrusts and factors influencing them in detail (Blanco‐Sacristan et al., 2019; Karnieli, 1997; Karnieli et al., 1996; Karnieli & Tsoar, 1995; Lehnert et al., 2018; O'Neill, 1993; Potter & Weigand, 2018; Rodriguez‐Caballero et al., 2015; Roman et al., 2019; Yamano et al., 2006). In situ hyperspectral data spectra have also been reported as useful for mapping lichens and cyanobacteria in a desert even it was not possible to separate in detail different components of the biocrusts (Ustin et al., 2009).

The first studies on mapping desert biocrusts using remote sensing data were conducted with Landsat‐3 MSS images in Southern Africa (Namib Desert) (Wessels & van Vuuren, 1986) and with aerial photography in the border zone of Israel‐Egypt (Karnieli & Tsoar, 1995). Later studies from arid areas have examined the potential of multispectral indices calculated from Sentinel‐2 MSI and Landsat 7 ETM+ to map biocrusts in China and South Africa (Chen et al., 2005; Rodriguez‐Caballero et al., 2022; Wang et al., 2022). In addition, in the advent of new spaceborne hyperspectral missions, the applicability of airborne hyperspectral data in monitoring dryland biocrusts has been assessed in Spain and South Africa (e.g., Rodriguez‐Caballero, Escribano, et al., 2017; Rodriguez‐Caballero, Paul, et al., 2017; Weber et al., 2008). Overall, both multi‐ and hyperspectral indices have shown potential in mapping biocrusts. However, a comparison of multi‐ and hyperspectral indices indicated that often high spectral resolution data would be needed for distinguishing biocrusts from the other surface materials (Rodriguez‐Caballero, Escribano, et al., 2017).

### 5.6 Lichens as part of animal habitat monitoring

A key motivation for mapping changes in lichen cover in northern areas of Fennoscandia, Canada, and the United States has been related to monitoring summer and winter pastures (grazing grounds) of reindeer and caribou—these animals feed on lichens. Through estimates of lichen cover, remote sensing can also provide a tool for participatory management for reindeer herders and their stakeholders (Sandstrom et al., 2003). The earliest studies in this specific context were conducted in the 1980s using Landsat‐5 TM images to map areas where lichen cover had changed due to reindeer grazing in northern Fennoscandia (Johansen & Tømmervik, 1990; Väre et al., 1996). To date, medium resolution satellite data mainly from Landsat sensors (5 TM, 7 ETM+ and 8 OLI) have been used for estimating lichen biomass (e.g., Colpaert et al., 2003, Colpaert & Kumpula, 2012; Erlandsson et al., 2023; Silva et al., 2019) or volume (Falldorf et al., 2014). However, more commonly, lichen cover has been mapped using Landsat (e.g., Gilichinsky et al., 2011; Macander et al., 2020; Nelson et al., 2013; Nordberg & Allard, 2014; Theau et al., 2005; Theau & Duguay, 2004) or SPOT‐5 (Gilichinsky et al., 2011) data. High spatial resolution satellite data have not been widely used, except for KOMPSAT‐3 data in a single study to map lichen cover and biomass for a site in Canada (Hillman & Nielsen, 2020). Different types of statistical and deep learning methods (e.g., spectral indices, generalized linear mixed‐effect models, enhancement‐classification, neural networks) and semiphysical methods (spectral mixture analysis) have been tested for estimating lichen cover or biomass, all relying on the special spectral features of lichens, which enable separating them from the surrounding green vegetation (see Section 5.2., and Figure 1).

Data collected by sensors onboard unoccupied aerial vehicles (UAV) have recently emerged also in lichen mapping. Lichen cover has been estimated for caribou areas in Canada and the United States based on UAV data using random forest models (Macander et al., 2020), various machine learning methods (He et al., 2021) and neural networks (Jozdani, Chen, Chen, Leblanc, Lovitt, et al., 2021a, Jozdani, Chen, Chen, Leblanc, Prevost, et al., 2021b; Richardson et al., 2021). While UAV data cannot be used to cover fully the extensive areas where reindeer and caribou herd, it can serve as part of a multiscale remote sensing framework which incorporates satellite and ground reference data as well as UAV data.

In addition to lichen mapping related to the caribou and reindeer grazing areas, Landsat‐based environmental variables, including lichen cover, have also been tested as part of developing denning habitat models for polar bears by Richardson et al., 2005. Based on their modeling, polar bears have been observed to den close to well‐drained, lichen‐dominated areas, which appear “brighter” than the surrounding vegetation in remote sensing data.

### 5.7 Lichens as part of spaceborne land cover and vegetation mapping in high latitudes

Lichens are included as a separate land surface class (or environmental variable) in approximately 40 articles related to vegetation monitoring in high latitudes (Fennoscandia, Canada, the United States, Antarctica, Russia) using multispectral satellite data. In these studies, lichens or descriptors of their coverage have often not been the sole focus of the research but rather one variable of interest among many, and only some papers report results or maps specifically on areas covered by lichens.

Separating lichens from their surrounding vegetation in optical remote sensing data in sub‐Arctic, Arctic, and tundra environments is based on the highly different spectral profile of, for example, common *Cladonia* lichens from other land surface types present (Rautiainen et al., 2007). Lichens have been included in the analyses of coarse spatial resolution mapping of northern vegetation and its phenology using AVHRR (e.g., Gamon et al., 2013) and MODIS data (e.g., Jia et al., 2009). More common data sources for mapping or monitoring lichens among other land surface types have been medium spatial resolution Landsat (e.g., Beck et al., 2015; Casanovas et al., 2015; Gilichinsky et al., 2011; Johansen & Karlsen, 2005; Macander et al., 2017; Nill et al., 2022; Orndahl et al., 2022; Smith et al., 2015; Tømmervik et al., 2003, 2004) and Sentinel‐2 sensors (e.g., Sotille et al., 2020). High spatial resolution satellite data, on the other hand, have been tested using Quickbird and WorldView sensors (e.g., Juutinen et al., 2017; Langford et al., 2016; Waser et al., 2007). To date, hyperspectral satellite (Huemmrich et al., 2013) and airborne data (Kuusinen et al., 2023) have been used clearly less than multispectral data to map lichens even though they have shown promising results for separating lichens from other land surface classes.

### 5.8 Spectral data on lichens in energy and carbon budget modeling

Land surface types (including vegetation and lichens) have a central role in the climate system as changes in their cover fractions alter the radiative and nonradiative properties of Earth (Duveiller et al., 2018). While model‐based studies have been extensively used to investigate the land–climate interactions (e.g., Feddema et al., 2005), the representation of many biophysical properties (such as lichen cover) in land surface models is still incomplete (e.g., de Noblet‐Ducoudré et al., 2012) even though lichens cover extensive areas in the high latitudes. Furthermore, the radiation balance of ecosystems, which have an abundant lichen or biocrust cover, such as subarctic tundra‐forests (Duguay et al., 1999) and drylands (Rodriguez‐Caballero et al., 2015), depends highly on surface wetness and phenology.

Even though the importance of including spectral remote sensing data of lichens in surface energy budget estimation was acknowledged already nearly four decades ago (Petzold & Goward, 1988), there are fewer than 10 articles which explicitly include spectral reflectance or albedo data of lichens in the context of energy or carbon budget modeling. Moreover, there is a lack of spatial data needed as input for land surface modeling: only predicted maps of bryophyte and lichen cover are available. Creating such maps is not trivial; for example, Rapalee et al. (2001) used Landsat TM to map moss and lichen cover in central Canada. Although they also used AVHRR data, lichen land cover could understandably not be mapped at 1 km spatial resolution. Furthermore, Porada et al. (2016) used a process‐based global land surface model JSBACH (Jena Scheme for Biosphere–Atmosphere Coupling in Hamburg) to predict lichen (and bryophyte) growth. Their results indicated that lichens and bryophytes have a significant impact on soil temperature in high latitudes, and that process‐based models are needed to reach realistic descriptions of their thermal properties. However, reliable maps on lichen cover based on remote sensing data would be required as input.

A concrete example of how remotely sensed data on the broadband spectral properties (albedo) of lichens can be applied in energy budget modeling is reported in the paper by Bernier et al. (2011), which looked at the climate change feedback that would result from the creation of open lichen woodlands in the closed‐canopy spruce forests. They investigated whether the increase in reflectance might compensate for the CO_2_ emissions from forest fires in terms of radiative forcing. The study was based on albedo estimates from multi‐year MODIS imagery and incoming solar radiation in combination with forest biomass estimates for eastern Canada. Their results showed that the net radiative forcing effect from closed‐canopy coniferous forests to open lichen woodlands would generate a cooling effect in the atmosphere.

Recently, a land surface model (E3SM Land Model, ELM) was configured for nine Arctic‐specific plant functional types (PFT, i.e., mosses, lichens, graminoids, and shrubs of different height classes and leaf habits) (Sulman et al., 2021). Even though this model configuration did not explicitly use spectral data on lichens, simulations through 2100 using the RCP8.5 climate scenario revealed that lichen‐dominated communities may be expected to gain less biomass compared to other PFTs. Further on, there is some evidence that shrubification (i.e., shift from a lichen heath to shrub vegetation) might lead to an average increase in atmospheric heating in arctic areas (Aartsma et al., 2021). Aartsma et al. (2021) found out that the daily net radiation of lichens was on average 26% lower than for shrubs during the growing season in alpine mountain area in southern Norway due to a higher albedo of the lichen heaths and to a larger longwave radiation loss. These results indicate a need for both spectral and broadband reflectance data on lichens in climate modeling applications.

## 6 CONCLUSIONS AND FUTURE PERSPECTIVES

Compared to many other fields of remote sensing, the number of scientific studies related to lichens is still fairly limited, fewer than 200 studies since the 1940s, and has been heavily biased towards lichen species and environments in northern Europe and North America. We have summarized the current situation and needs for future research related remote sensing of lichens in Table 3 and in the following text.

**TABLE 3:** A summary of the strengths, weaknesses, opportunities, and limitations of research related to remote sensing of lichens.

Current strengths	Future opportunities
Remote sensing of lichens has been motivated by very different types of applications Remote sensing data at a wide range of spatial resolutions have been applied to map lichens Lichens typically grow in open areas/ low vascular plant canopy cover which allows wide range of remote sensing instruments to be used for mapping and monitoring Species belonging to ~70 lichen species have had their spectral properties measured, and open spectral data are available for some species Lichens have a direct effect on surface albedo and (soil) surface insulation, and thus, spectral data on them are relevant for many research fields	Harmonized time series of medium resolution satellite data enable monitoring changes in lichen presence/absence and properties Using remotely‐sensed lichen properties as bioindicators in drought, erosion, animal habitat or climate change monitoring Applying hyperspectral data for identification of ground lichens species forming large mats Integrating spectral data on lichens in land surface modeling to improve climate change projections in northern latitudes

Current weaknesses	Limitations of remote sensing in lichen studies
Lichen species or biophysical/biochemical variables describing lichens or measurement conditions (incl. moisture levels) not commonly identified or reported in articles Spectral anisotropy of lichens poorly quantified compared to vascular plants, can have an impact on climate modeling through albedo effects Sample size or extent of field surveys to support remote sensing of lichens often limited A strong geographical bias towards lichen species growing in Europe and North America Use of hyperspectral airborne or satellite data to monitor lichens has been understudied Lichen mapping accuracies not reported in a consistent or comparable way in the studies	Many lichen species grow in mixed formations leading to difficulties identifying species or species‐specific properties in remote sensing data Growth of lichens occurs very slowly, and can be difficult to detect from remote sensing data

The spectral properties of large numbers of lichen species have been measured in laboratory or field settings (Tables 1 and 2), yet these data are not commonly openly available (except Kuusinen et al., 2022, 2023), mainly because the measurements have been primarily conducted before the open science movement. Nearly all spectral measurements of lichens have been conducted in a nadir or near‐nadir viewing geometry, and thus the reflectance anisotropy of lichens is still relatively poorly quantified compared to plants. This can have, for example, an impact of the applications of hemispherically integrated radiometric quantities (albedo) of ground lichens in, for example, surface energy budget modeling.

Overall, multispectral remote sensing dominates the literature in nearly all applications related to lichens, and the use of hyperspectral airborne or satellite data to retrieve lichen properties has been tested only in a few case studies. Currently, lichen cover, biomass, and volume are the most commonly estimated variables from remote sensing data but new and forthcoming hyperspectral satellite missions (e.g., CHIME, PRISMA, EnMAP) may open up possibilities for retrieving also other biophysical and biochemical properties of lichen mats. Identification of lichen species for remote biodiversity monitoring is, however, complicated by the mixed growth patterns of lichens, that is, not many species form large, monospecific mats on the ground but grow tightly together.

Besides using remote sensing data to map lichens and their biophysical and biochemical properties, an understanding of the spectral properties of lichens can have other significant impacts on advancement of science. For example, integrating lichens either as their own “PFT” to land surface models, energy budget calculation at a large scale will be a key improvement to projections of climate change in the boreal and Arctic regions because the spectral properties of lichens differ significantly from vegetation.

We conclude that lichens, although small organisms and frequently overlooked in the remote sensing community, present intriguing challenges for developing remote sensing methods and applications.

## AUTHOR CONTRIBUTIONS

**Miina Rautiainen:** Conceptualization (equal); data curation (equal); formal analysis (equal); investigation (equal); resources (lead); supervision (lead); writing – original draft (equal). **Nea Kuusinen:** Conceptualization (equal); investigation (equal); visualization (equal); writing – original draft (equal). **Titta Majasalmi:** Conceptualization (equal); investigation (equal); visualization (equal); writing – original draft (equal).

## CONFLICT OF INTEREST STATEMENT

The authors declare that they have no known competing financial interests or personal relationships that could have appeared to influence the work reported in this paper.

## Supporting information


Appendix S1

## Data Availability

Data sharing is not applicable to this article as no new original research data were created in this study. The list of articles that supports this study is available in the supplementary material of this article.

## References

[ece311110-bib-0001] Aartsma, P. , Asplund, J. , Odland, A. , Reinhardt, S. , & Renssen, H. (2021). Microclimatic comparison of lichen heaths and shrubs: Shrubification generates atmospheric heating but subsurface cooling during the growing season. Biogeosciences, 18, 1577–1599. 10.5194/bg-18-1577-2021

[ece311110-bib-0002] Ager, C. , & Milton, N. (1987). Spectral reflectance of lichens and their effects on the reflectance of rock substrates. Geophysics, 52(7), 839–1015.

[ece311110-bib-0003] Bechtel, R. , Rivard, B. , & Sanchez‐Azofeifa, A. (2002). Spectral properties of foliose and crustose lichens based on laboratory experiments. Remote Sensing of Environment, 82, 389–396.

[ece311110-bib-0004] Beck, I. , Ludwig, R. , Bernier, M. , Levesque, E. , & Boike, J. (2015). Assessing permafrost degradation and land cover changes (1986–2009) using remote sensing data over Umiujaq, sub‐Arctic Quebec. Permafrost and Periglacial Processes, 26, 129–141.

[ece311110-bib-0005] Bernes, C. , Bråthen, K. A. , Forbes, B. C. , Speed, J. , & Moen, J. (2015). What are the impacts of reindeer/caribou (*Rangifer tarandus* L.) on arctic and alpine vegetation? A systematic review. Environmental Evidence, 4, 4.

[ece311110-bib-0006] Bernier, P. , Desjardins, R. L. , Karimi‐Zindashty, Y. , Worth, D. , Beaudoin, A. , Luo, Y. , & Wang, S. (2011). Boreal lichen woodlands: A possible negative feedback to climate change in eastern North America. Agricultural and Forest Meteorology, 151(4), 521–528.

[ece311110-bib-0007] Blanco‐Sacristan, J. , Panigada, C. , Tagliabue, G. , Gentili, R. , Colombo, R. , de Guevara, M. L. , Maestre, F. T. , & Rossini, M. (2019). Spectral diversity successfully estimates the alpha‐diversity of biocrust‐forming lichens. Remote Sensing, 11, 2942. 10.3390/rs11242942

[ece311110-bib-0008] Bubier, J. L. , Rock, B. N. , & Crill, P. M. (1997). Spectral reflectance measurements of boreal wetland and forest mosses. Journal of Geophysical Research – Atmospheres, 102(D24), 29483–29494.

[ece311110-bib-0009] Casanovas, P. , Black, M. , Fretwell, P. , & Convey, P. (2015). Mapping lichen distribution on the Antarctic peninsula using remote sensing, lichen spectra and photographic documentation by citizen scientists. Polar Research, 34, 25633.

[ece311110-bib-0010] Chen, J. , Zhang, M. Y. , Wang, L. , Shimazaki, H. , & Tamura, M. (2005). A new index for mapping lichen‐dominated biological soil crusts in desert areas. Remote Sensing of Environment, 96, 165–175. 10.1016/j.rse.2005.02.011

[ece311110-bib-0011] Colpaert, A. , & Kumpula, J. (2012). Detecting changes in the state of reindeer pastures in northernmost Finland, 1995–2005. Polar Record, 48(SI1), 74–82.

[ece311110-bib-0012] Colpaert, A. , Kumpula, J. , & Nieminen, M. (2003). Reindeer pasture biomass assessment using satellite remote sensing. Arctic, 56(2), 147–158.

[ece311110-bib-0013] Conti, M. , & Cecchetti, G. (2001). Biological monitoring: Lichens as bioindicators of air pollution assessment – A review. Environmental Pollution, 114(3), 471–492. 11584645 10.1016/s0269-7491(00)00224-4

[ece311110-bib-0014] de Noblet‐Ducoudré, N. , Boisier, J. P. , Pitman, A. , Bonan, G. B. , Brovkin, V. , Cruz, F. , & Voldoire, A. (2012). Determining robust impacts of land‐use‐induced land cover changes on surface climate over North America and Eurasia: Results from the first set of LUCID experiments. Journal of Climate, 25(9), 3261–3281.

[ece311110-bib-0015] Duguay, C. , Rouse, W. , Lafleur, P. , & Boudreau, L. D. (1999). Radiation balance of wetland tundra at northern treeline estimated from remotely sensed data. Climate Research, 13(1), 77–90.

[ece311110-bib-0016] Duveiller, G. , Hooker, J. , & Cescatti, A. (2018). The mark of vegetation change on Earth's surface energy balance. Nature Communications, 9, 679. 10.1038/s41467-017-02810-8 PMC5820346 29463795

[ece311110-bib-0017] Erlandsson, R. , Arneberg, M. K. , Tømmervik, H. , Finne, E. A. , Nilsen, L. , & Bjerke, J. W. (2023). Feasibility of active handheld NDVI sensors for monitoring lichen ground cover. Fungal Ecology, 63, 101233. 10.1016/j.funeco.2023.101233

[ece311110-bib-0018] Falldorf, T. , Strand, O. , Panzacchi, M. , & Tømmervik, H. (2014). Estimating lichen volume and reindeer winter pasture quality from Landsat imagery. Remote Sensing of Environment, 140, 573–579.

[ece311110-bib-0019] Feddema, J. J. , Oleson, K. W. , Bonan, G. B. , Mearns, L. O. , Buja, L. E. , Meehl, G. A. , & Washington, W. M. (2005). The importance of land‐cover change in simulating future climates. Science, 310(5754), 1674–1678. 10.1126/science.1118160 16339443

[ece311110-bib-0020] Feng, J. , Rivard, B. , Rogge, D. , & Sanchez‐Azofeifa, A. (2013). The longwave infrared (3‐14 μm) spectral properties of rock encrusting lichens based on laboratory spectra and airborne SEBASS imagery. Remote Sensing of Environment, 131, 173–181.

[ece311110-bib-0021] Ferrenberg, S. , Reed, S. C. , Belnap, J. , & Schlesinger, W. H. (2015). Climate change and physical disturbance cause similar community shifts in biological soil crusts. Proceedings of the National Academy of Sciences of the United States of America, 112(39), 12116–12121. 26371310 10.1073/pnas.1509150112 PMC4593113

[ece311110-bib-0022] Feuerer, T. , & Hawksworth, D. L. (2007). Biodiversity of lichens, including a world‐wide analysis of checklist data based on Takhtajan's floristic regions. Biodiversity and Conservation, 16(1), 85–98.

[ece311110-bib-0023] Gamon, J. , Huemmrich, K. , Stone, R. , & Tweedie, C. (2013). Spatial and temporal variation in primary productivity (NDVI) of coastal Alaskan tundra: Decreased vegetation growth following earlier snowmelt. Remote Sensing of Environment, 129, 144–153.

[ece311110-bib-0024] Garty, J. , Kloog, N. , Cohen, Y. , Wolfson, R. , & Karnieli, A. (1997). The effect of air pollution on the integrity of chlorophyll, spectral reflectance response, and on concentrations of nickel, vanadium, and sulfur in the lichen Ramalina duriaei (De not.) Bagl. Environmental Research, 74(2), 174–118. 9339231 10.1006/enrs.1997.3727

[ece311110-bib-0025] Garty, J. , Kloog, N. , Wolfson, R. , Cohen, Y. , Karnieli, A. , & Avni, A. (1997). The influence of air pollution on the concentration of mineral elements, on the spectral reflectance response and on the production of stress‐ethylene in the lichen Ramalina duriaei. New Phytologist, 137(4), 587–597.

[ece311110-bib-0026] Garty, J. , Tamir, O. , Hassid, I. , Eshel, A. , Cohen, Y. , Karnieli, A. , & Orlovsky, L. (2001). Photosynthesis, chlorophyll integrity, and spectral reflectance in lichens exposed to air pollution. Journal of Environmental Quality, 30(3), 884–893. 11401277 10.2134/jeq2001.303884x

[ece311110-bib-0027] Garty, J. , Cohen, Y. , Kloog, N. , & Karnieli, A. (1997). Effects of air pollution on cell membrane integrity, spectral reflectance and metal and sulfur concentrations in lichens. Environmental Toxicology and Chemistry, 16(7), 1396–1402.

[ece311110-bib-0028] Garty, J. , Karnieli, A. , Wolfson, R. , Kunin, P. , & Garty‐Spitz, R. (1997). Spectral reflectance and integrity of cell membranes and chlorophyll relative to the concentration of airborne mineral elements in a lichen. Physiologia Plantarum, 101(2), 257–264.

[ece311110-bib-0029] Gauslaa, Y. (1984). Heat resistance and energy budget in different Scandinavian plants. Ecography, 7(1), 5–6.

[ece311110-bib-0030] Gilichinsky, M. , Sandstrom, P. , Reese, H. , Kivinen, S. , Moen, J. , & Nilsson, M. (2011). Mapping ground lichens using forest inventory and optical satellite data. International Journal of Remote Sensing, 32(2), 455–472.

[ece311110-bib-0031] Granlund, L. , Keski‐Saari, S. , Kumpula, T. , Oksanen, E. , & Keinänen, M. (2018). Imaging lichen water content with visible to mid‐wave infrared (400‐5500 nm) spectroscopy. Remote Sensing of Environment, 216, 301–310.

[ece311110-bib-0032] He, L. , Chen, W. , Leblanc, S. , Lovitt, J. , Arsenault, A. , Schmelzer, I. , Fraser, R. , Latifovic, R. , Sun, L. , Prevost, C. , White, P. , & Pouliot, D. (2021). Integration of multi‐scale remote sensing data for reindeer lichen fractional cover mapping in eastern Canada. Remote Sensing of Environment, 267, 112731.

[ece311110-bib-0033] Hillman, A. , & Nielsen, S. (2020). Quantification of lichen cover and biomass using field data, airborne laser scanning and high spatial resolution optical data – A case study from a Canadian boreal pine forest. Forests, 11(6), 682.

[ece311110-bib-0034] Huemmrich, K. , Gamon, J. , Tweedie, C. , Campbell, P. , Landis, D. , & Middleton, E. M. (2013). Arctic tundra vegetation functional types based on photosynthetic physiology and optical properties. IEEE Journal of Selected Topics in Applied Earth Observations and Remote Sensing, 6(2), 265–275.

[ece311110-bib-0035] Jacobberger, P. (1989). Reflectance characteristics and surface processes in stabilized dune environments. Remote Sensing of Environment, 28, 287–295.

[ece311110-bib-0036] Jia, G. , Epstein, H. , & Walker, D. (2009). Vegetation greening in the Canadian Arctic related to decadal warming. Journal of Environmental Monitoring, 11(12), 2231–2238. 20024021 10.1039/b911677j

[ece311110-bib-0037] Johansen, B. , & Karlsen, S. R. (2005). Monitoring vegetation changes on Finnmarksvidda, Northern Norway, using Landsat MSS and Landsat TM/ETM plus satellite images. Phytocoenologia, 35(4), 969–984. 10.1127/0340-269X/2005/0035-0969

[ece311110-bib-0038] Johansen, B. , & Tømmervik, H. (1990). Mapping winter grazing areas for reindeer in Finmark county, northern Norway, using Landsat 5‐TM data. 10th Annual International Symposium on Geoscience and Remote Sensing, p. 613–606. 10.1109/IGARSS.1990.688564

[ece311110-bib-0039] Jozdani, S. , Chen, D. , Chen, W. , Leblanc, S. , Lovitt, J. , He, L. , Fraser, R. , & Johnson, B. (2021). Evaluating image normalization via GANs for environmental mapping: A case study of lichen mapping using high‐resolution satellite imagery. Remote Sensing, 13(24), 5035.

[ece311110-bib-0040] Jozdani, S. , Chen, D. , Chen, W. , Leblanc, S. , Prevost, C. , Lovitt, J. , He, L. , & Johnson, B. (2021). Leveraging deep neural networks to map caribou lichen in high‐resolution satellite images based on a small‐scale, noisy UAV‐derived map. Remote Sensing, 13(14), 2658.

[ece311110-bib-0041] Juutinen, S. , Virtanen, T. , Kondratyev, V. , Laurila, T. , Linkosalmi, M. , Mikola, J. , Nyman, J. , Rasanen, A. , Tuovinen, J. , & Aurela, M. (2017). Spatial variation and seasonal dynamics of leaf‐area index in the arctic tundra‐implications for linking ground observations and satellite images. Environmental Research Letters, 12, 095002.

[ece311110-bib-0042] Kaasalainen, S. , & Rautiainen, M. (2005). Hot spot reflectance signatures of common boreal lichens. Journal of Geophysical Research: Atmospheres, 110(D20102), 1–10. 10.1029/2005JD005834

[ece311110-bib-0043] Karnieli, A. (1997). Development and implementation of spectral crust index over dune sands. International Journal of Remote Sensing, 18(6), 1207–1220.

[ece311110-bib-0044] Karnieli, A. , Shachak, M. , Tsoar, H. , Zaady, E. , Kaufman, Y. , Danin, A. , & Porter, W. (1996). The effect of microphytes on the spectral reflectance of vegetation in semiarid regions. Remote Sensing of Environment, 57, 88–96.

[ece311110-bib-0045] Karnieli, A. , & Tsoar, H. (1995). Spectral reflectance of biogenic crust developed on desert dune sand along the Israel‐Egypt border. International Journal of Remote Sensing, 16(2), 369–374.

[ece311110-bib-0046] Korpela, I. (2008). Mapping of understory lichens with airborne discrete‐return LiDAR data. Remote Sensing of Environment, 112(10), 3891–3897.

[ece311110-bib-0047] Krinov, E. L. (1947). Spectral reflectance properties of natural formations. Laboratoriia Aerometodov, Moscow, USSR. Technical Translation for National Research Council of Canada, no. NRC‐TT‐439. Translator: Belkov, G. in 1953. https://nrc‐publications.canada.ca/eng/view/ft/?id=8e981414‐eadd‐4cea‐85c6‐1579dd8f6181

[ece311110-bib-0048] Kushida, K. , Kim, Y. , Tanaka, N. , & Fukuda, M. (2004). Remote sensing of net ecosystem productivity based on component spectrum and soil respiration observation in a boreal forest, interior Alaska. Journal of Geophysical Research, 109(D06108), 1–11.

[ece311110-bib-0049] Kuusinen, N. , Hovi, A. , & Rautiainen, M. (2023). Estimation of boreal forest floor lichen cover using hyperspectral airborne and field data. Silva Fennica, 57(1), 22014.

[ece311110-bib-0050] Kuusinen, N. , Juola, J. , Karki, B. , Stenroos, S. , & Rautiainen, M. (2020). A spectral analysis of common boreal ground lichen species. Remote Sensing of Environment, 247, 111955. 32943799 10.1016/j.rse.2020.111955 PMC7371186

[ece311110-bib-0051] Kuusinen, N. , Juola, J. , Karki, B. , Stenroos, S. , & Rautiainen, M. (2022). Reflectance spectra of common boreal ground lichen species. Dataset. 10.17632/k482pn3gp4.1 PMC7371186 32943799

[ece311110-bib-0052] Laakso, K. , Rivard, B. , & Rogge, D. (2016). Enhanced detection of gossans using hyperspectral data: Example from the cape Smith Belt of northern Quebec, Canada. ISPRS Journal of Photogrammetry and Remote Sensing, 114, 137–150.

[ece311110-bib-0053] Langford, Z. , Kumar, J. , Hoffman, F. , Norby, R. , Wullschleger, S. , Sloan, V. , & Iversen, C. (2016). Mapping Arctic plant functional type distributions in the barrow environmental observatory using WorldView‐2 and LiDAR datasets. Remote Sensing, 8(9), 733.

[ece311110-bib-0054] Larson, D. W. (1987). The absorption and release of water by lichens. Bibliotheca lichenologica, 25, 351–360.

[ece311110-bib-0055] Lehnert, L. W. , Jung, P. , Obermeier, W. A. , Budel, B. , & Bendix, J. (2018). Estimating net photosynthesis of biological soil crusts in the Atacama using hyperspectral remote sensing. Remote Sensing, 10(891), 1–17. 10.3390/rs10060891

[ece311110-bib-0056] Li, L. , Solana, C. , Canters, F. , Chan, J. C. W. , & Kervyn, M. (2015). Impact of environmental factors on the spectral characteristics of lava surfaces: Field spectrometry of basaltic lava flows on Tenerife, Canary Islands, Spain. Remote Sensing, 7, 16986–17012. 10.3390/rs71215864

[ece311110-bib-0057] Macander, M. , Frost, G. , Nelson, P. , & Swingley, C. (2017). Regional quantitative cover mapping of tundra plant functional types in Arctic Alaska. Remote Sensing, 9(10), 1024.

[ece311110-bib-0058] Macander, M. , Palm, E. , Frost, G. , Herriges, J. , Nelson, P. , Roland, C. , Russell, K. , Suitor, M. , Bentzen, T. , Joly, K. , Goetz, S. , & Hebblewhite, M. (2020). Lichen cover mapping for caribou ranges in interior Alaska and Yukon. Environmental Research Letters, 15, 055001.

[ece311110-bib-0059] Milos, B. , Josef, H. , Jana, M. , Katerina, S. , & Alica, K. (2018). Dehydration‐induced changes in spectral reflectance indices and chlorophyll fluorescence of Antarctic lichens with different thallus color, and intrathalline photobiont. Acta Physiologiae Plantarum, 40, 177. 10.1007/s11738-018-2751-3

[ece311110-bib-0060] Moeslund, J. , Zlinszky, A. , Ejrnaes, R. , Brunbjerg, A. , Bocher, P. , Svenning, J. , & Normand, S. (2019). Light detection and ranging explains diversity of plants, fungi, lichens, and bryophytes across multiple habitats and large geographic extent. Ecological Applications, 29(5), e01907. 10.1002/eap.1907 31002436 PMC6852470

[ece311110-bib-0061] Morison, M. , Cloutis, E. , & Mann, P. (2014). Spectral unmixing of multiple lichen species and underlying substrate. International Journal of Remote Sensing, 35(2), 478–492.

[ece311110-bib-0062] Nash, T. (2008). Lichen biology. Cambridge University Press, ix + 486.

[ece311110-bib-0063] Nelson, P. R. , Roland, C. , Macander, M. J. , & McCune, B. (2013). Detecting continuous lichen abundance for mapping winter caribou forage at landscape spatial scales. Remote Sensing of Environment, 137, 43–54.

[ece311110-bib-0064] Neta, T. , Cheng, Q. , Bello, R. L. , & Hu, B. (2010). Lichens and mosses moisture content assessment through high‐spectral resolution remote sensing technology: A case study of the Hudson Bay Lowlands, Canada. Hydrological Processes, 24(18), 2617–2628.

[ece311110-bib-0065] Neta, T. , Cheng, Q. , Bello, R. L. , & Hu, B. (2011). Development of new spectral reflectance indices for the detection of lichens and mosses moisture content in the Hudson Bay Lowlands, Canada. Hydrological Processes, 25(6), 933–944.

[ece311110-bib-0066] Nill, L. , Grunberg, I. , Ullmann, T. , Gessner, M. , Boike, J. , & Hostert, P. (2022). Arctic shrub expansion revealed by Landsat‐derived multitemporal vegetation cover fractions in the Western Canadian Arctic. Remote Sensing of Environment, 281, 113228.

[ece311110-bib-0067] Nordberg, M. L. , & Allard, A. (2014). A remote sensing methodology for monitoring lichen cover. Canadian Journal of Remote Sensing, 28(2), 262–274. 10.5589/m02-026

[ece311110-bib-0068] O'Neill, A. L. (1993). Reflectance spectra of microphytic soil crusts in semiarid Australia. Remote Sensing Letters, 15(3), 675–681.

[ece311110-bib-0069] Orndahl, K. , Ehlers, L. , Herriges, J. , Pernick, R. , Hebblewhite, M. , & Goetz, S. (2022). Mapping tundra ecosystem plant functional type cover, height, and aboveground biomass in Alaska and northwest Canada using unmanned aerial vehicles. Arctic Science, 8, 1165–1180.

[ece311110-bib-0070] Paatero, J. , Dauvalter, V. , Derome, J. , Lehto, J. , Pasanen, J. , Vesala, T. , Miettinen, J. , Makkonen, U. , Kyrö, E.‐M. , Jernström, J. , Isaeva, L. , & Derome, K. (2008). Effects of Kola air pollution on the environment in the western part of the Kola peninsula and Finnish Lapland – Final report. Finnish Meteorological Institute Reports 2008:6. (ISBN 978‐951‐697‐686‐3).

[ece311110-bib-0071] Peltoniemi, J. , Kaasalainen, S. , Naranen, J. , Rautiainen, M. , Stenberg, P. , Smolander, H. , Smolander, S. , & Voipio, P. (2005). BRDF measurement of understory vegetation in pine forests: Dwarf shrubs, lichen, and moss. Remote Sensing of Environment, 94(3), 343–354.

[ece311110-bib-0072] Petzold, D. E. , & Goward, S. N. (1988). Reflectance spectra of subarctic lichens. Remote Sensing of Environment, 24(3), 481–492.

[ece311110-bib-0073] Porada, P. , Ekici, A. , & Beer, C. (2016). Effects of bryophyte and lichen cover on permafrost soil temperature at large scale. The Cryosphere, 10, 2291–2315. 10.5194/tc-10-2291-2016

[ece311110-bib-0074] Potter, C. , & Weigand, J. (2018). Imaging analysis of biological soil crusts to understand surface heating properties in the Mojave Desert of California. Catena, 170, 1–9. 10.1016/j.catena.2018.05.033

[ece311110-bib-0075] Rapalee, G. , Steyaert, L. T. , & Hall, F. G. (2001). Moss and lichen cover mapping at local and regional scales in the boreal forest ecosystem of central Canada. Journal of Geophysical Research: Atmospheres, 106(D24), 33551–33563.

[ece311110-bib-0076] Rautiainen, M. , Suomalainen, J. , Mõttus, M. , Stenberg, P. , Voipio, P. , Peltoniemi, J. , & Manninen, T. (2007). Coupling forest canopy and understory reflectance in the Arctic latitudes of Finland. Remote Sensing of Environment, 110(3), 332–343.

[ece311110-bib-0077] Rees, W. , & Williams, M. (1997). Monitoring changes in land cover induced by atmospheric pollution in the Kola Peninsula, Russia, using Landsat‐MSS data. International Journal of Remote Sensing, 18(8), 1703–1723.

[ece311110-bib-0078] Rees, W. G. , Tutubalina, O. V. , & Golubeva, E. (2004). Reflectance spectra of subarctic lichens between 400 and 2400 nm. Remote Sensing of Environment, 90(3), 281–292.

[ece311110-bib-0079] Regan, S. , Matwichuk, L. , Cloutis, E. , Goltz, D. , & Mann, P. (2016). Potential signatures of heavy metal complexes in lichen reflectance spectra. International Journal of Remote Sensing, 37(11), 2621–2640.

[ece311110-bib-0080] Richardson, E. , Stirling, I. , & Hik, D. (2005). Polar bear (*Ursus maritimus*) maternity denning habitat in western Hudson Bay: A bottom‐up approach to resource selection functions. Canadian Journal of Zoology, 83, 6–870.

[ece311110-bib-0081] Richardson, G. , Leblanc, S. , Lovitt, J. , Rajaratnam, K. , & Chen, W. (2021). Leveraging AI to estimate Caribou lichen in UAV Orthomosaics from ground photo datasets. Drones, 5(3), 99.

[ece311110-bib-0082] Rivard, B. , Zhang, J. , Feng, J. , & Sanchez‐Azofeifa, G. A. (2009). Remote predictive lithologic mapping in the Abitibi Greenstone Belt, Canada, using airborne hyperspectral imagery. Canadian Journal of Remote Sensing, 35(19), S95–S105.

[ece311110-bib-0083] Rodriguez‐Caballero, E. , Escribano, P. , Olehowski, C. , Chamizo, S. , Hill, J. , Canton, Y. , & Weber, B. (2017). Transferability of multi‐ and hyperspectral optical biocrust indices. ISPRS Journal of Photogrammetry and Remote Sensing, 126, 94–107.

[ece311110-bib-0084] Rodriguez‐Caballero, E. , Knerr, T. , & Weber, B. (2015). Importance of biocrusts in dryland monitoring using spectral indices. Remote Sensing of Environment, 170, 32–39.

[ece311110-bib-0085] Rodriguez‐Caballero, E. , Paul, M. , Tamm, A. , Caesar, J. , Budel, B. , Escribano, P. , Hill, J. , & Weber, B. (2017). Biomass assessment of microbial surface communities by means of hyperspectral remote sensing data. Science of the Total Environment, 586, 1287–1297. 28236481 10.1016/j.scitotenv.2017.02.141

[ece311110-bib-0086] Rodriguez‐Caballero, E. , Reyes, A. , Kratz, A. , Caesar, J. , Guirado, E. , Schmiedel, U. , Escribano, P. , Fiedler, S. , & Weber, B. (2022). Effects of climate change and land use intensification on regional biological soil crust cover and composition in southern Africa. Geoderma, 406, 115508. 10.1016/j.geoderma.2021.115508

[ece311110-bib-0087] Rollin, E. M. , Milton, E. J. , & Roche, P. (1994). The influence of weathering and lichen cover of the reflectance spectral of granitic rocks. Remote Sensing of Environment, 50, 194–199.

[ece311110-bib-0088] Roman, J. R. , Rodriguez‐Caballero, E. , Rodriguez‐Lozano, B. , Roncero‐Ramos, B. , Chamizon, S. , Aguila‐Carricondo, P. , & Canton, Y. (2019). Spectral response analysis: An indirect and non‐destructive methodology for the chlorophyll quantification of biocrusts. Remote Sensing, 11, 1350. 10.3390/rs11111350

[ece311110-bib-0089] Salehi, S. , Rogge, D. , Rivard, B. , Heincke, B. H. , & Fensholt, R. (2017). Modeling and assessment of wavelength displacements of characteristic absorption features of common rock forming minerals encrusted by lichens. Remote Sensing of Environment, 199, 78–92.

[ece311110-bib-0090] Sandstrom, P. , Pahlen, T. G. , Edenius, L. , Tømmervik, H. , Hagner, O. , Hemberg, L. , Olsson, H. , Baer, K. , Stenlund, T. , Brandt, L. G. , & Egberth, M. (2003). Conflict resolution by participatory management: Remote sensing and GIS as tools for communicating land‐use needs for reindeer herding in northern Sweden. Ambio, 32(8), 557–567. 15049353 10.1579/0044-7447-32.8.557

[ece311110-bib-0091] Satterwhite, M. B. , Henley, J. P. , & Carney, J. M. (1985). Effects of lichens on the reflectance spectra of granitic rock surfaces. Remote Sensing of Environment, 18, 105–112.

[ece311110-bib-0092] Silva, J. A. , Nielsen, S. E. , Lamb, C. , Hague, C. , & Boutin, S. (2019). Modelling lichen abundance for woodland caribou in a fire‐driven boreal landscape. Forests, 10, 1–23. 10.3390/f10110962

[ece311110-bib-0093] Sipman, H. , & Aptroot, A. (2001). Where are the missing lichens? Mycological Research, 105, 1433–1439.

[ece311110-bib-0094] Smith, A. , Hill, M. , & Zhang, Y. (2015). Estimating ground cover in the mixed prairie grassland of southern Alberta using vegetation indices related to physiological function. Canadian Journal of Remote Sensing, 41(1), 51–66.

[ece311110-bib-0095] Solheim, I. , Engelsen, O. , Hosgood, B. , & Andreoli, G. (2000). Measurement and modeling of the spectral and directional reflection properties of lichen and moss canopies. Remote Sensing of Environment, 72(1), 78–94.

[ece311110-bib-0096] Sotille, M. , Bremer, U. , Vieira, G. , Velho, L. , Petsch, C. , & Simoes, J. (2020). Evaluation of UAV and satellite‐derived NDVI to map maritime Antarctic vegetation. Applied Geography, 25, 102322.

[ece311110-bib-0097] Steiner, D. , & Gutermann, D. (1966). Russian data on spectral reflectance of vegetation, soil and rock types: A final technical report. European Research Office: United States Army (contract number: DA‐91‐59i‐EUC‐386 3 / 01‐652‐0006). Juris Druck + Nierlag Zurich, 233 p. https://apps.dtic.mil/sti/pdfs/AD0815418.pdf

[ece311110-bib-0098] Sulman, B. N. , Salmon, V. G. , Iversen, C. M. , Breen, A. L. , Yuan, F. , & Thornton, P. E. (2021). Integrating arctic plant functional types in a land surface model using above‐and belowground field observations. Journal of Advances in Modeling Earth Systems, 13(4), e2020MS002396.

[ece311110-bib-0099] Theau, J. , & Duguay, C. R. (2004). Lichen mapping in the summer range of the George River caribou herd using Landsat TM imagery. Canadian Journal of Remote Sensing, 6, 867–881.

[ece311110-bib-0100] Theau, J. , Peddle, D. R. , & Duguay, C. R. (2005). Mapping lichen in a caribou habitat of northern Quebec, Canada, using an enhancement‐classification method and spectral mixture analysis. Remote Sensing of Environment, 94(2), 232–243.

[ece311110-bib-0101] Tømmervik, H. , Hogda, K. , & Solheim, L. (2003). Monitoring vegetation changes in Pasvik (Norway) and Pechenga in Kola peninsula (Russia) using multitemporal Landsat MSS/TM data. Remote Sensing of Environment, 83(3), 370–388.

[ece311110-bib-0102] Tømmervik, H. , Johansen, B. , & Pedersen, J. (1995). Monitoring the effects of air‐pollution on terrestrial ecosystems in Varanger (Norway) and Nikel‐Pechenga (Russia) using remote‐sensing. Science of the Total Environment, 160–161, 753–767.

[ece311110-bib-0103] Tømmervik, H. , Johansen, B. , Tombre, I. , Thannheiser, D. , Hogda, K. , Gaare, E. , & Wielgolaski, F. E. (2004). Vegetation changes in the Nordic mountain birch forest: The influence of grazing and climate change. Arctic, Antarctic, and Alpine Research, 36(3), 323–332.

[ece311110-bib-0104] Tømmervik, H. , Johansen, M. , Pedersen, J. , & Guneriussen, T. (1998). Integration of remote sensed and in‐situ data in an analysis of the air pollution effects on terrestrial ecosystems in the border areas between Norway and Russia. Environmental Monitoring and Assessment, 49, 51–85.

[ece311110-bib-0105] Ustin, S. L. , Valko, P. G. , Kefauver, S. C. , Santos, M. J. , Zimpfer, J. F. , & Smith, S. D. (2009). Remote sensing of biological soil crust under simulated climate change manipulations in the Mojave Desert. Remote Sensing of Environment, 113, 317–328. 10.1016/j.rse.2008.09.013

[ece311110-bib-0106] Väre, H. , Ohtonen, R. , & Mikkola, K. (1996). The effect and extent of heavy grazing by reindeer in oligotrophic pine heaths in northeastern Fennoscandia. Ecography, 19, 245–253.

[ece311110-bib-0107] Virtanen, T. , Mikkola, K. , Patova, E. , & Nikula, A. (2002). Satellite image analysis of human caused changes in the tundra vegetation around the city of Vorkuta, north‐European Russia. Environmental Pollution, 120(3), 647–658. 12442788 10.1016/s0269-7491(02)00186-0

[ece311110-bib-0108] Vitikainen, O. (2001). Great discoveries in bryology and lichenology: William Nylander (1822‐1899) and lichen chemotaxonomy. The Bryologist, 104(2), 262–267.

[ece311110-bib-0109] Wang, Z. , Wu, B. , Zhang, M. , Zeng, H. , Yang, L. , Tian, F. , Ma, Z. , & Wu, H. (2022). Indices enhance biological soil crust mapping in sandy and desert lands. Remote Sensing of Environment, 278, 113078. 10.1016/j.rse.2022.113078

[ece311110-bib-0110] Waser, L. , Kuechler, M. , Schwarz, M. , Ivits, E. , Stofer, S. , & Scheidegger, C. (2007). Prediction of lichen diversity in an UNESCO biosphere reserve – Correlation of high resolution remote sensing data with field samples. Environmental Modeling & Assessment, 12(4), 315–328.

[ece311110-bib-0111] Weber, B. , Olehowski, C. , Knerr, T. , Hill, J. , Deutschewitz, K. , Wessels, D. C. J. , Eitel, B. , & Budel, B. (2008). A new approach for mapping of Biological Soil Crusts in semidesert areas with hyperspectral imagery. Remote Sensing of Environment, 112, 2187–2201. 10.1016/j.rse.2007.09.014

[ece311110-bib-0112] Wessels, D. C. J. , & Van Vuuren, D. R. J. (1986). Landsat imagery – Its possible use in mapping the distribution of major lichen communities in the Namib Desert, south West Africa. Madoqua, 14, 369–373.

[ece311110-bib-0113] Yamano, H. , Chen, J. , Zhang, Y. , & Tamura, M. (2006). Relating photosynthesis of biological soil crusts with reflectance: Preliminary assessment based on a hydration experiment. International Journal of Remote Sensing, 27(24), 5393–5399.

[ece311110-bib-0114] Zhang, J. K. , Rivard, B. , & Sanchez‐Azofeifa, A. (2004). Derivative spectral unmixing of hyperspectral data applied to mixtures of lichen and rock. IEEE Transactions on Geoscience and Remote Sensing, 42(9), 1934–1940.

[ece311110-bib-0115] Zhang, J. K. , Rivard, B. , & Sanchez‐Azofeifa, A. (2005). Spectral unmixing of normalized reflectance data for the deconvolution of lichen and rock mixtures. Remote Sensing of Environment, 95, 57–66.

